# Sulfiredoxin-1 exerts anti-apoptotic and neuroprotective effects against oxidative stress-induced injury in rat cortical astrocytes following exposure to oxygen-glucose deprivation and hydrogen peroxide

**DOI:** 10.3892/ijmm.2015.2205

**Published:** 2015-05-08

**Authors:** YUNCHUAN ZHOU, YANG ZHOU, SHANSHAN YU, JINGXIAN WU, YANLIN CHEN, YONG ZHAO

**Affiliations:** 1 Department of Pathology, Chongqing Medical University, Chongqing 400016, P.R. China; 2 Institute of Neuroscience, Chongqing Medical University, Chongqing 400016, P.R. China

**Keywords:** sulfiredoxin-1, astrocytes, apoptosis, neuroprotection

## Abstract

Sulfiredoxin 1 (Srxn1), an endogenous antioxidant protein, plays an important neuroprotective role in cerebral ischemia. However, the exact mechanisms of action of Srxn1 in cerebral ischemia have not yet been fully elucidated. Therefore, in the present study, rat primary cortical astrocytes transfected with a lentiviral vector encoding short hairpin RNA (shRNA) were exposed to oxygen-glucose deprivation (OGD) for 4 h or to 100 *μ*M hydrogen peroxide (H_2_O_2_) for 6 h, in order to construct an *in vitro* model of cerebral ischemia-induced damage. We found that following exposure to OGD or H_2_O_2_, the knockdown of Srxn1 resulted in a decrease in cell viability, as shown by MTS assay, an increase in cell damage, as shown by lactate dehydrogenase assay and an increase in cellular apoptosis, as shown by Hoechst 33342 staining and flow cytometry. Furthermore, we found that following exposure to OGD or H_2_O_2_, the knockdown of Srxn1 resulted in a decrease in mitochondrial transmembrane potential (Δψ_m_) as indicated by JC-1 staining, an increase in the cytoplasmic expression of cytochrome *c* (Cyt.C), caspase-3, caspase-9, poly(ADP-ribose) polymerase (PARP) and Bax protein at the protein level, but a decrease in the expression of the anti-apoptotic Bcl-2 protein; these effects were tightly associated with the mitochondrial apoptotic pathway. However, we found that there was no obvious change in the intracellular calcium ([Ca^2+^]i) levels and caspase-12 expression following the knockdown of Srxn1. Taken together, the results from the present study demonstrate that Srxn1 protects primary rat cortical astrocytes from OGD- or H_2_O_2_-induced apoptosis and that involves the activation of the mitochondrial apoptotic pathway, which suggests that Srxn1 may be a potential target in the treatment of cerebral ischemia.

## Introduction

Cerebral ischemia, particularly during the period of ischemia/reperfusion, involves complex biochemical mechanisms, and compared with other organs, brain tissue is more likely to produce free radicals and lipid peroxides; therefore, oxidative stress is likely to be the key factor responsible for the irreversible damage caused during cerebral ischemia/reperfusion (1). It has been confirmed that oxidative stress can affect glial cells, which are associated with neuronal death or a decrease in neuronal cell proliferation (2). At present, however, we have not acquired a satisfactory curative effect with regards to the application of exogenous antioxidants and anti-apoptotic agents. It is well known that the endogenous antioxidant defense system is crucial to the survival of nerve cells (3). Therefore, endogenous antioxidants and anti-apoptotic agents may provide an effective therapeutic intervention in ischemic brain injury. In addition, an improved understanding of the disease mechanisms is crucial for developing applicable treatment strategies.

Sulfiredoxin 1 (Srxn1), a central endogenous antioxidant protein belonging to the sulfiredoxin family of antioxidants, was initially characterized in yeast (4) and subsequently in mammalian cells (5) before it was reported to have an important function in neuroprotection (3). Srxn1 functions as one of the reactive oxygen species (ROS) management systems that counter cell oxidative stress-induced damage (6). It has been demonstrated that Srxn1 protects brain tissue from damage through the regulation of glutathionylation/deglutathionylation in Parkinson’s disease (PD) (7). The induction of Srxn1 expression has been shown to exert neuroprotective effects against hydrogen peroxide (H_2_O_2_)-induced cell death (8). The reduction of Srxn1 expression has been shown to lead to an increased sensitivity to oxidative stress, and this sensitivity was reduced by the overexpression of intracellular Srxn1 (6,9,10). In addition, it has been reported that the induction of the expression of Srxn1 contributes to neuroprotective ischemic preconditioning in response to oxygen-glucose deprivation (OGD) *in vitro* and following brief ischemic episodes *in vivo* (11). Moreover, Srxn1 can enter the mitochondria to maintain a balance between mitochondrial H_2_O_2_ production and elimination (12). Furthermore, Srxn1 may be a novel component in maintaining the balance between H_2_O_2_ production and elimination and protecting A549 cells or wild-type mouse embryonic fibroblasts (MEFs) from apoptosis (13). This may be related to the inhibition of the release of cytochrome *c* (Cyt.C) and the activation of caspase-9 and caspase-3 (13). Nevertheless, the specific function and related regulatory mechanisms of action of Srxn1 in cerebral ischemia and in damage induced by apoptosis have not been extensively investigated.

In our previous study, we demonstrated that the knockdown of Srxn1 resulted in an increased sensitivity to H_2_O_2_ in PC12 cells, indicating that Srxn1 was essential for antioxidant proteins (1). The aim of this study was to investigate the function of Srxn1 with respect to apoptosis in rat cortical astrocytes, as well as the potential protective mechanisms of action of Srxn1.

## Materials and methods

### Reagents

Glucose-high Dulbecco’s modified Eagle’s medium/F12 (DMEM/F12), glucose-free DMEM and fetal bovine serum (FBS) were purchased from Gibco (Grand Island, NY, USA); poly-L-lysine and H_2_O_2_ were from Sigma-Aldrich (Milan, Italy); 5,5′,6,6′-tetrachloro-1,1′,3,3′-tetraethylbenzimidazol-carbocyanine iodide (JC-1), Fluo-3, AM and Annexin V-FITC/propidium iodide (PI) were obtained from Molecular Probes, Invitrogen (Milan, Italy), and MTS was from Promega Corp. (Madison, WI, USA); Hank’s solution and trypsin were obtained from HyClone (Logan, UT, USA), and Hoechst 33342, phosphate-buffered saline (PBS) and penicillin/streptomycin (Pen/Strep) were from Beyotime (Shanghai, China). The lactate dehydrogenase (LDH) assay kit was purchased from Roche Molecular Biochemicals (Indianapolis, IN, USA).

### Primary culture of rat cortical astrocytes

All procedures were in accordance with the Guide for the Care and Use of Laboratory Animals adopted by the National Institutes of Health (Bethesda, MA, USA). Newborn Sprague-Dawley (SD) rats (day 0–1-old) were obtained from the Laboratory Animal Center, Chongqing Medical University, Chongqing, China. Cortical astrocytes from newborn SD rats were obtained as previously described (14,15). The cells in DMEM/F12 medium containing 10% FBS and 1% Pen/Strep were plated onto poly-L-lysine-coated 75 cm^2^ flasks at a density of 1.5×10^5^ cells/ml at 37°C in a incubator (Thermo 3111; Thermo Scientific, Waltham, MA, USA) containing 5% CO_2_ and 95% air. When firmly attached to the bottom of the flask, the cells were passaged. Tha astrocytes obtained using this procedure were then sub-cultured twice. Under these conditions, the astrocytes were contained at >90% as determined by glial fibrillary acidic protein (GFAP) staining.

### Transfection with short hairpin RNA (shRNA) against Srxn1

A Srxn1 lentivirus (LV2-nonGFP)-encoding shRNA and a negative control (NC) shRNA were prepared by Shanghai GenePharma (Shanghai, China), using the rat Srxn1 gene sequence NM_001047858 available in the NCBI database. The effective knockdown fragment sequence of shRNA against rat Srxn1 was CAT CCA CAC CAG ACT TGC AGT, and the sequence of the negative control shRNA against rat was TTC TCC GAA CGT GTC ACG T. The astrocytes were transfected with the shRNA according to the directions provided by the manufacturer and as described in the study by Liu *et al* (16). Briefly, after the culture medium was changed, 1 *μ*l of lenti-virus per 1×10^4^ cells was added to the cells, which were 60–80% confluent, for infection for approximately 24 h. The cells were then washed with PBS, and the culture medium was changed. Following transfection for approximately 72 h, the cells were ready for exposure to OGD or H_2_O_2_. The Srxn1 shRNA knockdown efficiency was confirmed by western blot analysis and reverse transcription-quantitative polymerase chain reaction (RT-qPCR) at approximately 72 h post-transfection.

### Cell groups and exposure to OGD or H_2_O_2_

The astrocytes were divided into the following 9 groups: i) the control group: astrocytes without any treatment; ii) the negative control (NC) group: astrocytes were transfected with negative control shRNA; iii) the sh-Srxn1 group: astrocytes were transfected with Srxn1 shRNA (sh-Srxn1); iv) the control + OGD group: astrocytes were exposed to OGD; v) the NC + OGD group: astrocytes were transfected with negative control shRNA followed by exposure to OGD; vi) the sh-Srxn1 + OGD group: astrocytes were transfected with Srxn1 shRNA followed by exposure to OGD; vii) the control + H_2_O_2_ group: astrocytes were exposed to H_2_O_2_; viii) the NC + H_2_O_2_ group: astrocytes were transfected with negative control shRNA followed by exposure to H_2_O_2_; and ix) the sh-Srxn1+H_2_O_2_ group: astrocytes were transfected with Srxn1 shRNA followed by exposure to H_2_O_2_.

OGD was performed according to a previously described method (15). Briefly, the control and the cells transfected with the shRNA for 72 h were exposed to OGD for 4 h, followed by oxygen-glucose reoxygenation for 24 h at 37°C. Subsequently, the cells were ready for further experiments. H_2_O_2_ was administered according to a previously described method (1). The control and the cells transfected with the shRNA for 72 h were exposed to H_2_O_2_ at an optimal concentration of 100 *μ*M for 6 h at 37°C; the cells were then ready for the following experiments.

### Western blot analysis

The cells were lysed with PRO-PREP™ liquid (Beyotime) in the presence of a protease inhibitor cocktail (Roche, Mannheim, Germany), and subsequently centrifuged at 12,000 rpm for 15 min at 4°C. The total cytoplasmic protein concentration was determined using a bicinchoninic acid (BCA) assay kit (Beyotime). Equal amounts (20 *μ*g) of total protein were separated by 16-6% sodium dodecyl sulfate-polyacrylamide gel (SDS-PAGE) gels and transferred onto polyvinylidene fluoride (PVDF) membranes (0.22 and 0.45 *μ*m; Millipore, Bedford, MA, USA). The membranes were blocked with 5% non-fat milk in TBST buffer for 1 h and then sequentially incubated with rabbit polyclonal anti-Srxn1 (1:400; bs-8329R; Bioss, Beijing, China), rabbit polyclonal anti-Cyt.C (1:1,000; ab92509; Abcam Ltd., Cambridge, UK), rabbit polyclonal anti-caspase-3 (1:500; sc-7148), mouse polyclonal anti-caspase-9 (1:400; sc-8355) (both from Santa Cruz Biotechnology, Inc., Santa Cruz, CA, USA), rabbit polyclonal anti-caspase-12 (1:400; bs-1105R; Bioss), rabbit polyclonal anti-poly(ADP-ribose) polymerase (1:800; ab6070; Abcam Ltd.), rabbit polyclonal anti-Bax (1:400; sc-6236), rabbit polyclonal anti-Bcl-2 (1:400; sc-492) (both from Santa Cruz Biotechnology, Inc., Santa Cruz, CA, USA) and mouse monoclonal anti-β-actin (1:1,000; AP0060; Bioworld Technology, Inc., St. Louis Park, MN, USA) as the primary antibodies overnight at 4°C. After being washed, the bound antibodies were detected by incubation for 1.5 h at room temperature with goat anti-rabbit or goat anti-mouse HRP-conjugated secondary antibodies (1:2,000; BS10350 and BS12478, respectively; Bioworld Technology, Inc.). The membranes were visualized by using the electro-chemiluminescence (ECL) detection system and quantified using Quantity One image analysis software (both from Bio-Rad, Hercules, CA, USA).

### RNA extraction and RT-qPCR

Total RNA was extracted using RNAiso Plus (Takara Biotechnology Co., Ltd., Dalian, China) according to the manufacturer’s instructions. The concentration and purity of the RNA were measured with a spectrophotometer (U-3010; Hitachi Co., Tokyo, Japan). Subsequently, cDNA from the total RNA was obtained using the PrimeScript™ RT reagent kit (Perfect Real-Time) (Takara Biotechnology Co., Ltd.). The amplification was carried out using the Thermal Cycler miniopticon real-time PCR system (Bio-Rad) in a 25 *μ*l reaction mixture containing 2 *μ*l of diluted cDNA templates, 1 *μ*l of each primer and 12.5 *μ*l of 2X SYBR^®^ Premix Ex Taq™ (Takara Biotechnology Co., Ltd.). The PCR conditions were as follows: 95°C for 10 sec, followed by 39 cycles consisting of 95°C for 5 sec, 60°C for 15 sec and 72°C for 15 sec, and melt curve at 72°C 5 sec, followed by 95°C keep. The sequences of the specific primers used in this study were as follows: Srxn1 forward, 5′-CCC AAG GCG GTG ACT ACT AC-3′ and reverse, 5′-GGC AGG AAT GGT CTC TCT CTG TG-3′; β-actin forward, 5′-CAC CCG CGA GTA CAA CCT TC-3′ and reverse, 5′-CCC ATA CCC ACC ATC ACA CC-3′. β-actin was used to normalize the expression levels of each sample. The reaction of each sample was performed in triplicate. Data were calculated using 2^−ΔΔCt^ method, as previously described (17,18) with Bio-Rad CFX Manager software (Bio-Rad).

### 3-(4,5-Dimethylthiazol-2-yl)-5-(3-carboxymethoxyphenyl)-2- (4-sulfophenyl)-2H-tetrazolium (MTS) assay for cell viability

The number of viable cells was determined using the CellTiter 96 AQueous One Solution Cell Proliferation Assay according to the manufacturer’s instructions (Promega, Fitchburg, WI, USA). In brief, the treated cells (1×10^4^ cells/well) were added to 20 *μ*l of CellTiter 96 AQueous One Solution Reagent. The plate was then incubated at 37°C for 3 h. The absorbance was measured at 490 nm using a microtiter plate reader (Bio-Rad).

### LDH assay

The release of LDH was measured using the LDH assay kit according to manufacturer’s instructions (Roche Molecular Biochemicals). Following treatment, the nutrient solution was collected and transferred to new 96-well plates, then mixed with the 100 *μ*l reaction solution provided in the kit. The optical density was measured at 490 nm 30 min using a microplate reader (Bio-Rad).

### Hoechst staining

Apoptotic morphology was measured using Hoechst 33342 staining as previously described (15). Briefly, the treated cells on coverslips (approximately 1×10^5^ cells/coverslip) were fixed with 4% paraformaldehyde for 20 min at 37°C. After washing, the cells were mounted with Hoechst 33342 at room temperature for 15 min. Fluorescent images were acquired using a fluorescence microscope (Olympus BX51; Olympus, Tokyo, Japan). Nuclear shrinkage, chromatin condensation and nuclear fragmentation were the criteria for identifying an apoptotic cell.

### Flow cytometric analysis

Apoptosis analyses were performed as previously described (19), using Annexin V-FITC and propidium iodide (PI) double staining. The treated cells were harvested and diluted in 100 *μ*l of 1X Annexin V binding buffer per assay and incubated with Annexin V-FITC and PI for 15 min in the dark at room temperature. Subsequently, 400 *μ*l of 1X binding buffer were added. The stained cells were immediately analyzed by flow cytometry (FCM). During apoptosis, phosphatidylserine is translocated from the inner to the outer leaflet of the plasma membrane where it can be detected by Annexin V conjugates. For each sample, at least 1×10^5^ cells were analyzed by applying CellQuest™ Pro Analysis software (BD Biosciences, San Jose, CA, USA). The cytogram of the 4 quadrants was used to distinguish normal (Annexin V^−^/PI^−^), early apoptotic (Annexin V^+^/PI^−^), late apoptotic (Annexin V^+^/PI^+^) and necrotic cells (Annexin V^−^/PI^+^). The sum of the early and late apoptotic cells represented the total apoptosis.

### Measurement of mitochondrial transmembrane potential (Δψ_m_)

To measure the Δψ_m_, JC-1, a lipophilic cation sensitive fluorescent probe for Δψ_m_, was used according to the manufacturer’s instructions (Molecular Probes) and as described in the study by Han *et al* (20). The cells were washed and incubated with 10 *μ*g/ml JC-1 at 37°C for 30 min. After removing JC-1 and washing, images were captured by Laser Scanning Confocal Microscopy (Nikon TiE; Nikon, Tokyo, Japan) with both red and green channels. A total of 6 random, non-adjacent fields in each group were used for statistical analysis. IPP 6.0 software was used to measure the average fluorescence intensity of the red and green fluorescence in each group. The Δψ_m_ level is represented by the JC-1 fluorescence ratio, which was calculated as the ratio of average fluorescence intensity of red/green.

### Measurements of intracellular calcium ([Ca^2+^]i) levels

To monitor changes in [Ca^2+^]i levels, Fluo-3 AM was used according to the manufacturer’s instructions (Invitrogen). The cells were washed and incubated with 5 *μ*M of the Ca^2+^ indicator, Fluo-3/AM, at 37°C for 60 min. After removing Ca^2+^ and washing, changes in [Ca^2+^]i levels (Fluo-3 fluorescence intensity) were measured by Laser Scanning Confocal Microscopy (Nikon TiE; Nikon). A total of 6 random, non-adjacent fields in each group were used for statistical analysis.

### Statistical analysis

All the experiments were performed at least 3 times and the data are presented as the means ± SEM. Data were analyzed by one-way ANOVA followed by a post hoc Tukey’s test. Statistical analysis was performed using SPSS 17.0 software (SPSS, Inc., Chicago, IL, USA) and a value of P<0.05 was considered to indicate a statistically significant difference.

## Results

### Evaluation of the efficiency of Srxn1 shRNA following exposure to OGD or H_2_O_2_

**Figure 1:**
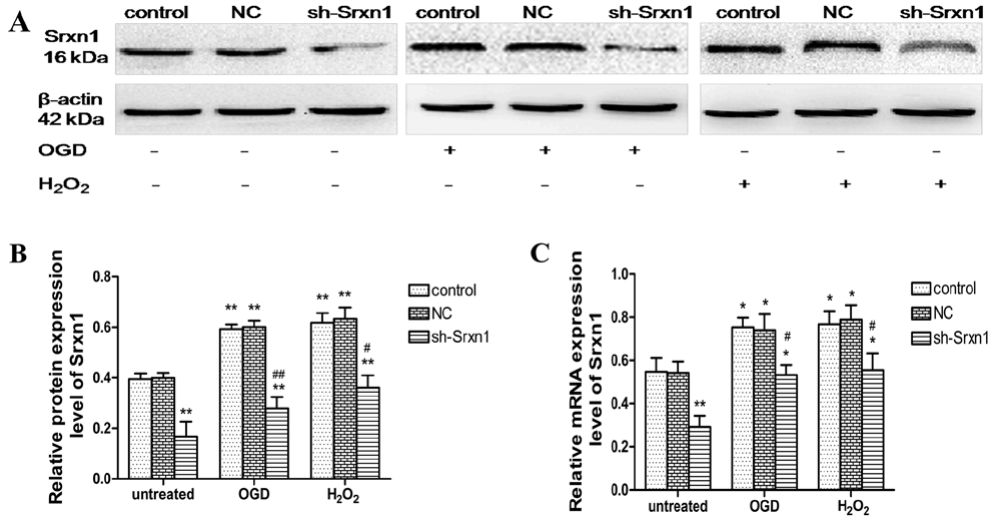
Evaluation of the efficiency of short hairpin RNA (shRNA) against sulfiredoxin 1 (Srxn1) following exposure to oxygen-glucose deprivation (OGD) or hydrogen peroxide (H_2_O_2_). (A and B) Srxn1 protein expression was measured by western blot analysis. (C) Srxn1 mRNA expression was determined by RT-qPCR. The results showed that Srxn1 protein and mRNA expression were decreased by shRNA Srxn1 knockdown and that sensitivity to OGD or H_2_O_2_ was increased. The exposure to astrocytes to OGD or H_2_O_2_ resulted in a significant increase in Srxn1 protein and mRNA expression, and Srxn1 knockdown prevented this increase. Data are the means ± SEM. ^*^P<0.05 and ^**^P<0.01 vs. controls (untreated cells); ^#^P<0.05 and ^##^P<0.01 vs. control + OGD (or H_2_O_2_) group; n=4. NC, negative control.

The inhibition of Srxn1 expression in the presence of OGD or H_2_O_2_ was confirmed by western blot analysis (Fig. 1A and B) and RT-qPCR (Fig. 1C). Compared with the control group, Srxn1 knockdown resulted in a ~61% decrease in protein and a ~47% decrease in Srxn1 mRNA expression (Fig. 1). The exposure of the cells to OGD (or H_2_O_2_) resulted in a significant increase in Srxn1 protein and mRNA expression, and Srxn1 knockdown prevented this increase (sh-Srxn1 + OGD group and sh-Srxn1 + H_2_O_2_ group), compared with the control + OGD (or H_2_O_2_) group (P<0.05 and P<0.01). These results suggest that Srxn1 plays an important role in protecting astrocytes from damage induced by exposure to OGD or H_2_O_2_.

### Knockdown of Srxn1 decreases cell viability and enhances cell damage following exposure to OGD or H_2_O_2_

**Figure 2:**
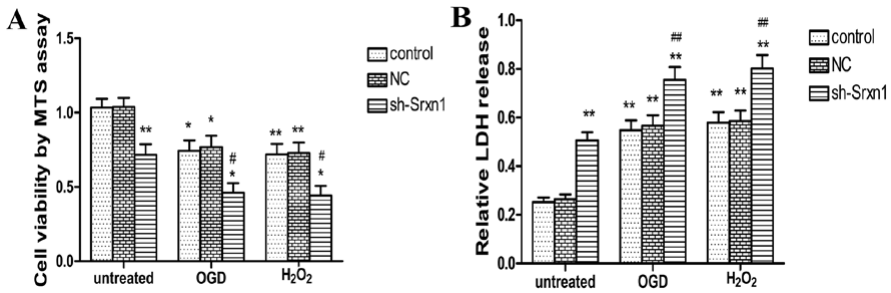
Knockdown of sulfiredoxin 1 (Srxn1) decreases cell viability and increases cell damage following exposure to oxygen-glucose deprivation (OGD) or hydrogen peroxide (H_2_O_2_). (A) Cell viability was determined by MTS assay. Exposure to OGD or H_2_O_2_ decreased cell viability. Following exposure to OGD or H_2_O_2_, the knockdown of Srxn1 promoted the reduction in cell viability. Values are the means ± SEM; ^*^P<0.05, ^**^P<0.01 vs. controls (untreated cells); ^#^P<0.05 vs. control + OGD (or H_2_O_2_) group. n=6 from 3 independent experiments. (B) Cell damage was assessed by measuring the lactate dehydrogenase (LDH) levels. Exposure to OGD or H_2_O_2_ increased cell damage. Following exposure to OGD or H_2_O_2_, the knockdown of Srxn1 increased cell damage even more significantly. n=3 from 3 independent experiments. Values are the means ± SEM; ^**^P<0.01 vs. controls (untreated cells); ^##^P<0.01 vs. control + OGD (or H_2_O_2_) group. NC, negative control.

Following exposure to OGD or H_2_O_2_, cell viability was determined by MTS assay (Fig. 2A). Compared with the control group, following exposure to OGD or H_2_O_2_, cell viability was decreased by ~26% in the control + OGD group and by 27% in the control + H_2_O_2_ group. Following the knockdown of Srxn1, cell viability decreased more significantly (sh-Srxn1 + OGD group and sh-Srxn1 + H_2_O_2_ group) compared with the control + OGD (or H_2_O_2_) group (P<0.05). We also determined astrocyte damage by LDH assay (Fig. 2B). Exposure to OGD (or H_2_O_2_) resulted in an increase in the release of LDH (P<0.05), and following exposure to OGD or H_2_O_2_, the knockdown of Srxn1 resulted in the release of LDH increasing more significantly compared with the control + OGD (or H_2_O_2_) group (P<0.01; Fig. 2B). These results indicate that Srxn1 may protect astrocytes from OGD- or H_2_O_2_-induced damage.

### Knockdown of Srxn1 increases apoptosis following exposure to OGD or H_2_O_2_

**Figure 3:**
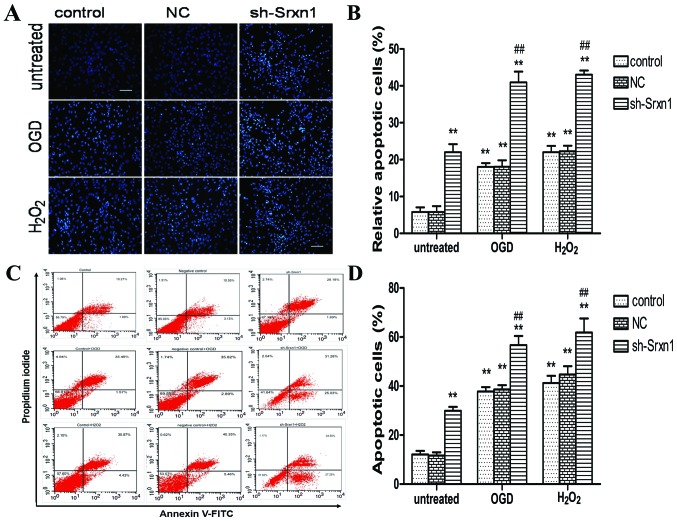
Knockdown of sulfiredoxin 1 (Srxn1) increases apoptosis following exposure to oxygen-glucose deprivation (OGD) or hydrogen peroxide (H_2_O_2_). (A and B) Apoptosis was assessed by Hoechst 33342 staining and visualized by fluorescence. Exposure to OGD or H_2_O_2_ increased cell apoptosis. Following exposure to OGD or H_2_O_2_, the knockdown of Srxn1 increased apoptosis increased more significantly. Scale bar, 20 *μ*m. Results are expressed as the means ± SEM; ^**^P<0.01 vs. controls (untreated cells); ^##^P<0.01 vs. control + OGD (or H_2_O_2_) group, n=4. (C and D) Flow cytometric analysis of apoptosis and necrosis. (C) Typical representative dot plots of the distribution of normal (Annexin V^−^/PI^−^), early apoptotic (Annexin V^+^/PI^−^), late apoptotic (Annexin V^+^/PI^+^) and necrotic (Annexin V^−^/PI^+^) cells. (D) Late-apoptotic and early-apoptotic astrocytes were calculated from the total astrocytes. Following exposure to OGD or H_2_O_2_, the knockdown of Srxn1 markedly increased apoptosis. Data are the means ± SEM; ^**^P<0.01 vs. controls (untreated cells); ^##^P<0.01 vs. control + OGD (or H_2_O_2_) group, n=4. NC, negative control (unreated cells).

Apoptosis was detected by combining microscopic analysis with Hoechst 33342 staining (Fig. 3A and B) and FCM (Fig. 3C and D). Compared with the control group, exposure to OGD (or H_2_O_2_) induced apoptosis (control + OGD group and control + H_2_O_2_ group) (P<0.01), and following the knockdown of Srxn1, the cell apoptotic rate increased more significantly (sh-Srxn1 + OGD group and sh-Srxn1 + H_2_O_2_ group) compared with the control + OGD (or H_2_O_2_) group (P<0.01; Fig. 3A and B). Annexin V-FITC/PI double staining revealed a significant increase in apoptotis by ~29% in the control + OGD group and by 31% in the control + H_2_O_2_ group, compared with the control group (P<0.01; Fig. 3C and D). In addition, following exposure to OGD or H_2_O_2_, the knockdown of Srxn1 resulted in an even more significant increase in apoptotis (P<0.01), compared with the control + OGD (or H_2_O_2_) group. Taken together, these results indicate that Srxn1 plays a vital role in protecting astrocytes from apoptosis induced by exposure to OGD (or H_2_O_2_).

### Knockdown of Srxn1 activates the mitochondrial apoptotic pathway following exposure to OGD or H_2_O_2_

**Figure 4:**
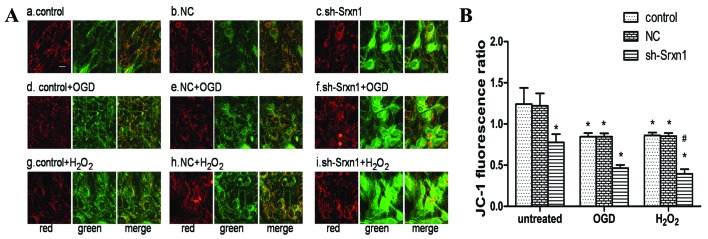
Changes in mitochondrial membrane potential (Δψ_m_) following exposure to oxygen-glucose deprivation (OGD) or hydrogen peroxide (H_2_O_2_) and the knockdown of sulfiredoxin 1 (Srxn1) based on JC-1 staining. (A) (Panels a and b) Laser scanning confocal microscopic images revealed that both green and red fluorescence were detected in the controls (untreated cells) and that the red fluorescence was stronger than the green fluorescence. (Panel c) The green fluorescence was stronger and the red fluorescence was weaker in the sh-Srxn1 group than the control group. (Panels f and i) After the knockdown of Srxn1 and exposure to OGD or H_2_O_2_, the green fluorescence became much stronger than that in the control + OGD (or H_2_O_2_) group. Scale bar, 100 *μ*m. (B) The JC-1 fluorescence ratio was calculated by the average optical fluorescence density ratio of red/green in each group. The JC-1 fluorescence ratio decreased by ~38% in the sh-Srxn1 group compared with that in the control group. The JC-1 fluorescence ratio in the sh-Srxn1 + OGD (or H_2_O_2_) group declined more significantly compared with that in the control + OGD (or H_2_O_2_) group. Data are the means ± SEM; ^*^P<0.05 vs. controls (untreated cells); ^#^P<0.05 vs. control + OGD (or H_2_O_2_) group, n=4. (NC, negative control).

**Figure 5:**
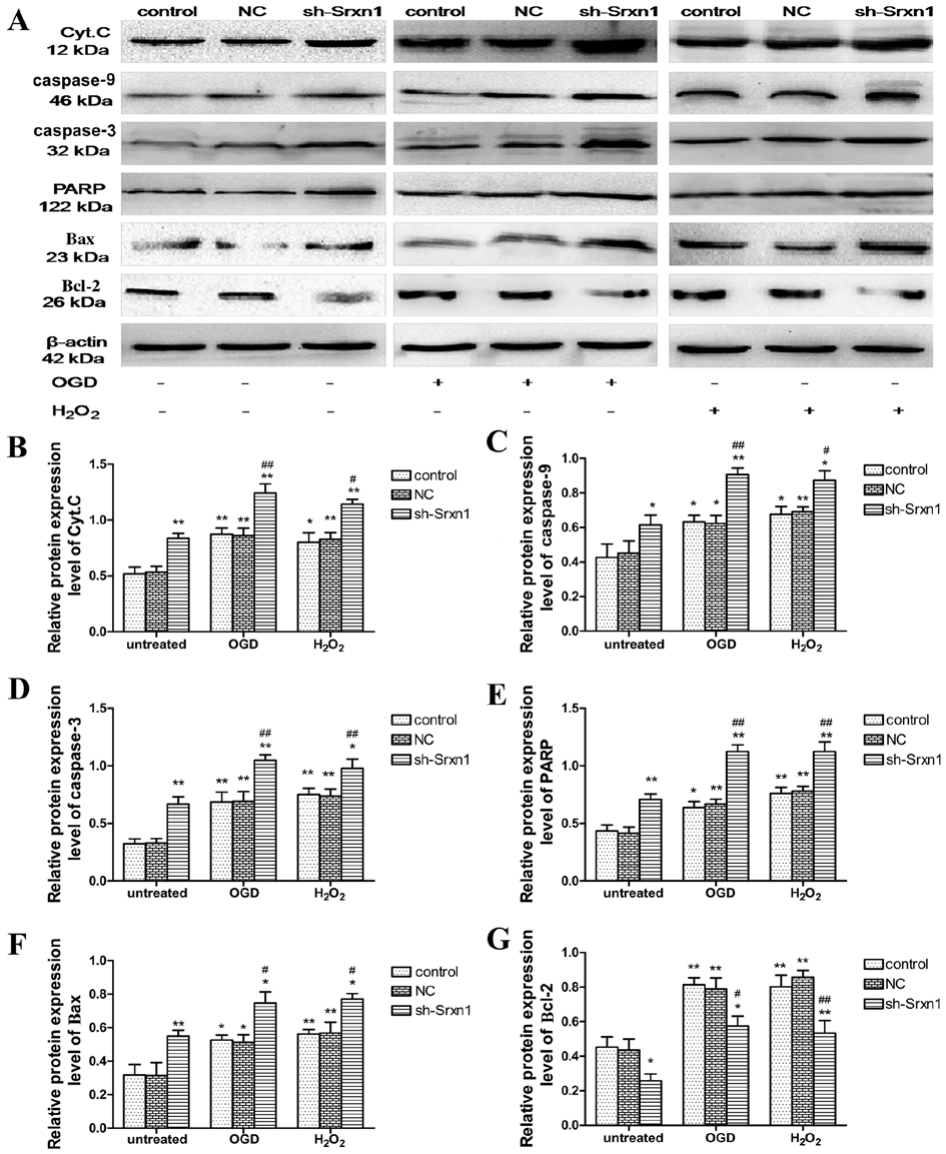
Effect of exposure to oxygen-glucose deprivation (OGD) or hydrogen peroxide (H_2_O_2_) and the knockdown of sulfiredoxin 1 (Srxn1) on the expression levels of mitochondrial apoptotic pathway-related proteins. Representative western blots data quantification of (A and B) cytochrome *c* (Cyt.C), (A and C) caspase-9, (A and D) caspase-3, (A and E) poly(ADP-ribose) polymerase (PARP), (A and F) Bax and (A and G) Bcl-2 expression in astrocytes. The results revealed that the levels of these proteins increased in response to exposure to OGD or H_2_O_2_. Following exposure to OGD or H_2_O_2_, Srxn1 knockdown promoted these expression levels, apart from Bcl-2 expression; the level of Bcl-2 decreased. Data are the means ± SEM; ^*^P<0.05 and ^**^P<0.01 vs. controls (untreated cells); ^#^P<0.05 and ^##^P<0.01 vs. control + OGD (or H_2_O_2_) group, n=4. NC, negative control.

To evaluate whether the mitochondrial apoptotic pathway was activated, we determined Δψ_m_ using JC-1 staining (Fig. 4), as well as the expression levels of some mitochondrial apoptotic pathway-related proteins by western blot analysis (Fig. 5). At a high membrane potential, JC-1 enters the mitochondria and forms aggregates that have a fluorescence of bright red, whereas JC-1 exists as a green fluorescence at a low potential (21). As shown in Fig. 4A, the untreated cell mitochondria exhibited a high Δψ_m_, as indicated by a bright, red fluorescence and a very low green fluorescence (Fig. 4A panels a and b). The knockdown of Srxn1 decreased Δψ_m_, and thus the green fluorescence increased significantly compared with the red fluorescence (Fig. 4A panel c). In addition, following exposure to OGD or H_2_O_2_, the green fluorescence was stronger than the red fluorescence [control + OGD (or H_2_O_2_) group], compared with the untreated control group (Fig. 4A panels d, e, g and h). Following exposure to OGD or H_2_O_2_, Srxn1 knockdown resulted in a further decrease in Δψ_m_, and thus the green fluorescence became much stronger and the red fluorescence became much weaker (Fig. 4A panels f and i). To quantify the level of Δψ_m_, the JC-1 fluorescence ratio was calculated using the average optical density ratio of red/green. The JC-1 ratio decreased by ~38% in the sh-Srxn1 group, by 32% in the control + OGD group and by 31% in the control + H_2_O_2_ group, compared with the untreated control group (P<0.05; Fig. 4B). Moreover, following exposure to OGD or H_2_O_2_, Srxn1 knockdown markedly decreased the JC-1 ratio in the sh-Srxn1 + OGD (or H_2_O_2_) group compared with the control + OGD (or H_2_O_2_) group (P<0.05), representing a dissipation of Δψ_m_. These data indicated that Srxn1 knockdown induced the dissipation of Δψ_m_ in the astrocytes following exposure to OGD or H_2_O_2_.

Compared with the control group, exposure to OGD or H_2_O_2_ increased the cytoplasmic release of Cyt.C, as well as the cytoplasmic levels of caspase-9, caspase-3, PARP, Bax and Bcl-2 proteins (P<0.05; Fig. 5). Following exposure to OGD or H_2_O_2_, Srxn1 knockdown promoted this increase in protein expression apart from the expression of Bcl-2; the expression level of Bcl-2 decreased (sh-Srxn1 + OGD group and sh-Srxn1 + H_2_O_2_ group) compared with the control + OGD (or H_2_O_2_) group (P<0.01 or P<0.05). These observations indicate that Srxn1 has an anti-apoptotic function in astrocytes following exposure to OGD or H_2_O_2_, which is related to the mitochondrial apoptotic pathway.

### Knockdown of Srxn1 does not affect endoplasmic reticulum stress pathway-mediated apoptosis following exposure to OGD or H_2_O_2_

**Figure 6:**
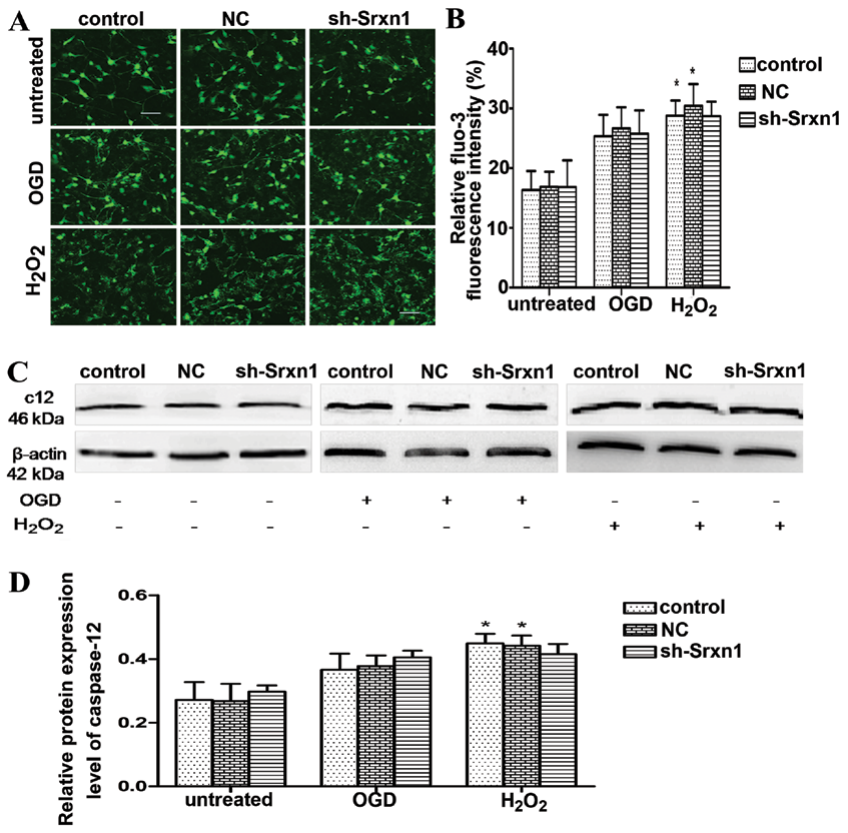
Effect of the knockdown of sulfiredoxin 1 (Srxn1) on endoplasmic reticulum stress pathway-mediated apoptosis. (A and B) Intracellular calcium ([Ca^2+^]i) levels were measured by laser scanning confocal microscopy. Images showed that green fluorescence had a fluo-3 fluorescence intensity. The stronger the fluorescence for [Ca^2+^]i, the higher the expression. Compared with the controls (untreated cells), the level of [Ca^2+^]i markedly increased in the control + oxygen-glucose deprivation (OGD) [or hydrogen peroxide (H_2_O_2_)] group, and the level of [Ca^2+^]i did not change significantly after the knockdown of Srxn1. Data are the means ± SEM; ^*^P<0.05 vs. controls (untreated cells), n=4; (C and D) Western blot analysis for caspase-12. Exposure to OGD or H_2_O_2_ increased the level of caspase-12, but it did not affect caspase-12 expression after the knockdown of Srxn1. Data are the means ± SEM; ^*^P<0.05 vs. controls (untreated cells), n=4.

In order to determine whether endoplasmic reticulum stress pathway-mediated apoptosis had occurred, we detected [Ca^2+^]i and caspase-12 levels based on Fluo-3 AM staining (Fig. 6A and B) and western blot analysis (Fig. 6C and D). Of note, we found that the levels of caspase-12 and [Ca^2+^]i increased following exposure to OGD or H_2_O_2_ compared with the control group; however, after the knockdown of Srxn1, these levels did not differ from those of the control + OGD (or H_2_O_2_) group. Hence, we deduced that the neuroprotective effects of Srxn1 are not related to endoplasmic reticulum stress pathway-mediated apoptosis.

## Discussion

In the present study, we provide evidence that Srxn1 exerts a protective effect against oxidative stress-induced brain injury and also provide insight into its mechanisms of action. Rat corital astrocytes were exposed to OGD and H_2_O_2_ in order to mimic oxidative stress-induced injury in cerebral ischemia and used shRNAs to knockdown Srxn1. We found that following exposure to OGD or H_2_O_2_, the knockdown of Srxn1 significantly increased cell death as determined by MTS and LDH assays, increased cell apoptosis as measured by Hoechst 33342 and Annexin V-FITC/PI staining. Following exposure to OGD or H_2_O_2_, the knockdown of Srxn1 promoted the activation of the mitochondrial apoptotic pathway, decreasing Δψ_m_ and the expression of the anti-apoptotic Bcl-2 protein in the cytoplasm, and increasing the cytoplasmic levels of Cyt.C, caspase-3, caspase-9, PARP and pro-apoptotic Bax proteins. We also demonstrated that the levels of [Ca^2+^]i and caspase-12, which are tightly linked with endoplasmic reticulum stress pathway-mediated apoptosis, were not altered after the knockdown of Srxn1. Taken together, these results indicate that Srxn1 plays a vital role in protecting astrocytes from apoptosis induced by exposure to OGD or H_2_O_2_. Srxn1 was found play a neuroprotective role partly by exerting anti-apoptosis effects associated with the mitochondrial apoptotic pathway.

Cerebral ischemia, particularly during the period of ischemia/reperfusion, involves complex pathophysiological and biochemical mechanisms, such as the generation of ROS, glutamate-mediated excitotoxicity, inflammation, calcium-activated proteolysis and apoptosis (22,23). It has been widely reported that apoptosis is the major cell death pathway following hypoxia-ischemia in developing brains (24–26). Within the central nervous system (CNS), astrocytes are the most abundant cells and play a role in synaptic transmission and plasticity, they transport nutrients and participate in neurotransmission (27–29). It has been demonstrated that astrocytes in primary culture undergo apoptosis following an ischemic insult (30,31). Furthermore, studies have indicated that oxidative stress and glial-derived ROS are critical for the apoptosis-induced selective loss of neurons (32,33). Moreover, astrocyte apoptosis may contribute to the pathogenesis of a number of acute and chronic neurodegenerative disorders, such as cerebral ischemia, Alzheimer’s disease and PD (34).

There are three main apoptotic signaling pathways that have been discovered: i) the mitochondrial pathway (intrinsic pathway) (35,36); ii) the death receptor-associated pathway (extrinsic pathway) (37); and iii) the endoplasmic reticulum-associated pathway (38,39). Currently, the apoptosis of the mitochondria and the endoplasmic reticulum pathway, which are endogenous organelles, are hotspot research topics. A variety of conditions can disturb the functions of the mitochondria or the endoplasmic reticulum and lead to mitochondrial or endoplasmic reticulum stress. These conditions include ischemia, hypoxia, exposure to free radicals, as well as others. When mitochondrial or endoplasmic reticulum stress conditions persist, the initiation of the apoptotic processes is promoted.

Under different pathological conditions, cell death is associated with or initiated by mitochondrial function impairment and dissipation of Δψ_m_ (40). Δψ_m_, which can modulate the release of Cyt.C by controlling membrane permeability, plays a crucial role in the terminal step of apoptosis (41,42). The depolarization of mitochondrial membrane potential induced by hypoxic-ischemic injury implicates the loss of Δψ_m_ in the brain. The loss of Δψ_m_ leads to mitochondrial membrane permeabilization, and thus the release of Cyt.C from the mitochondria into the cytoplasm, which binds and activates caspase-9, thereby resulting in the activation of other downstream caspases and, ultimately, caspase-3 (43), triggering apoptosis (41,42). Caspase-3 has been identified as a main executioner of the apoptotic response inside cells. Finally, activated caspase-3 cleaved effector proteins, including PARP and the induction of DNA fragmentation in the nucleus eventually lead to cell death (44,45). Bcl-2 family members are the arbiters of the mitochondrial apoptotic pathway and decide whether a cell lives or dies (46). This family includes anti-apoptotic (e.g., Bcl-2) and pro-apoptotic genes (e.g., Bax) (47,48). Bcl-2, an inhibitor of apoptosis, prevents neuronal apoptosis by blocking the destruction of the mitochondria and the subsequent release of Cyt.C and caspase activation (49). In this study, our results demonstrated that OGD or H_2_O_2_ increased cell death, including apoptosis, decreased Δψ_m_ and increased the subsequent cytosolic translocation of Cyt.C, thereby promoting downstream caspase activation, resulting in increased levels of caspase-9, caspase-3, PARP (the substrate of caspase-3), anti-apoptotic protein Bcl-2 and pro-apoptotic protein Bax in the cytoplasm. Following exposure to OGD or H_2_O_2_, Srxn1 knockdown promoted cell death, apoptosis, dissipation of Δψ_m_, and promoted an increase in protein expression apart from Bcl-2 expression (the expression of Bcl-2 decreased). These data indicated that the knockdown of Srxn1 promoted the activation of the mitochondrial apoptotic pathway following exposure to OGD or H_2_O_2_. Thus, the protective effects of Srxn1 are associated with the inhibition of the mitochondrial apoptotic pathway.

It has been reported that caspase-12-mediated apoptosis is a specific apoptotic pathway of the endoplasmic reticulum (39,50). Caspase-12 appears to be necessary for apoptosis induced by a variety of endoplasmic reticulum-directed pro-apoptotic signals (39). The cytoplasmic calcium [Ca^2+^]c signal has been shown to mediate the calpain/caspase-12-dependent apoptotic pathway primarily in cancer cells, and it has been shown that alterations in [Ca^2+^]c levels may potentially induce apoptotic cell death (51). In this study, exposure to OGD or H_2_O_2_ resulted in endoplasmic reticulum stress and thus incresaed the expression of caspase-12 and [Ca^2+^]i. However, after the knockdown of Srxn1, these levels were not altered. This suggests that the knockdown of Srxn1 does not affect endoplasmic reticulum stress pathway-mediated apoptosis.

In conclusion, the data from the present study demonstrate that Srxn1 exerts a protective effect against the oxidative stress-induced brain injury and that this effect is partly mediated through its anti-apoptosis effects, which are associated with the inhibition of the mitochondrial apoptotic pathway. The findings of this study suggest that Srxn1 may be a novel target for neuroprotective intervention in neurodegenerative diseases. We aim to continue this area of investigation in future *in vivo* studies using the chemical synthesis of RNA interference and the overexpression of Srxn1 to verify the association between Srxn1 and apoptosis.

## References

[1] Li Q, Yu S, Wu J, Zou Y, Zhao Y (2013). Sulfiredoxin-1 protects PC12 cells against oxidative stress induced by hydrogen peroxide. J Neurosci Res.

[2] Kinsner A, Pilotto V, Deininger S (2005). Inflammatory neurodegeneration induced by lipoteichoic acid from Staphylococcus aureus is mediated by glia activation, nitrosative and oxidative stress, and caspase activation. J Neurochem.

[3] Papadia S, Soriano FX, Leveille F (2008). Synaptic NMDA receptor activity boosts intrinsic antioxidant defenses. Nat Neurosci.

[4] Biteau B, Labarre J, Toledano MB (2003). ATP-dependent reduction of cysteine-sulphinic acid by S. cerevisiae sulphiredoxin. Nature.

[5] Rhee SG, Jeong W, Chang TS, Woo HA (2007). Sulfiredoxin, the cysteine sulfinic acid reductase specific to 2-Cys peroxiredoxin: its discovery, mechanism of action, and biological significance. Kidney Int Suppl.

[6] Vivancos AP, Castillo EA, Biteau B (2005). A cysteine-sulfinic acid in peroxiredoxin regulates H_2_O_2_-sensing by the antioxidant Pap1 pathway. Proc Natl Acad Sci USA.

[7] Findlay VJ, Tapiero H, Townsend DM (2005). Sulfiredoxin: a potential therapeutic agent?. Biomed Pharmacother.

[8] Soriano FX, Leveille F, Papadia S (2008). Induction of sulfiredoxin expression and reduction of peroxiredoxin hyperoxidation by the neuroprotective Nrf2 activator 3H-1,2-dithiole-3-thione. J Neurochem.

[9] Kang KW, Lee SJ, Kim SG (2005). Molecular mechanism of nrf2 activation by oxidative stress. Antioxid Redox Signal.

[10] Singh A, Ling G, Suhasini AN (2009). Nrf2-dependent sulfiredoxin-1 expression protects against cigarette smoke-induced oxidative stress in lungs. Free Radic Biol Med.

[11] Bell KF, Al-Mubarak B, Fowler JH (2011). Mild oxidative stress activates Nrf2 in astrocytes, which contributes to neuroprotective ischemic preconditioning. Proc Natl Acad Sci USA.

[12] Noh YH, Baek JY, Jeong W, Rhee SG, Chang TS (2009). Sulfiredoxin translocation into mitochondria plays a crucial role in reducing hyperoxidized peroxiredoxin III. J Biol Chem.

[13] Baek JY, Han SH, Sung SH (2012). Sulfiredoxin protein is critical for redox balance and survival of cells exposed to low steady-state levels of H_2_O_2_. J Biol Chem.

[14] Wu J, Li Q, Wang X (2013). Neuroprotection by curcumin in ischemic brain injury involves the Akt/Nrf2 pathway. PLoS One.

[15] Wu X, Zhao J, Yu S, Chen Y, Wu J, Zhao Y (2012). Sulforaphane protects primary cultures of cortical neurons against injury induced by oxygen-glucose deprivation/reoxygenation via anti-apoptosis. Neurosci Bull.

[16] Liu SB, Zhang N, Guo YY (2012). G-protein-coupled receptor 30 mediates rapid neuroprotective effects of estrogen via depression of NR2B-containing NMDA receptors. J Neurosci.

[17] Schmittgen TD, Livak KJ (2008). Analyzing real-time PCR data by the comparative C(T) method. Nat Protoc.

[18] Song S, Zhou J, He S (2013). Expression levels of microRNA-375 in pancreatic cancer. Biomed Rep.

[19] Lin CJ, Chen TH, Yang LY, Shih CM (2014). Resveratrol protects astrocytes against traumatic brain injury through inhibiting apoptotic and autophagic cell death. Cell Death Dis.

[20] Han F, Tao RR, Zhang GS (2011). Melatonin ameliorates ischemic-like injury-evoked nitrosative stress: Involvement of HtrA2/PED pathways in endothelial cells. J Pineal Res.

[21] Sung DK, Chang YS, Kang S, Song HY, Park WS, Lee BH (2010). Comparative evaluation of hypoxic-ischemic brain injury by flow cytometric analysis of mitochondrial membrane potential with JC-1 in neonatal rats. J Neurosci Methods.

[22] Zhao J, Kobori N, Aronowski J, Dash PK (2006). Sulforaphane reduces infarct volume following focal cerebral ischemia in rodents. Neurosci Lett.

[23] Chan PH (2001). Reactive oxygen radicals in signaling and damage in the ischemic brain. J Cereb Blood Flow Metab.

[24] Hossain MA (2008). Hypoxic-ischemic injury in neonatal brain: involvement of a novel neuronal molecule in neuronal cell death and potential target for neuroprotection. Int J Dev Neurosci.

[25] Kawamura M, Nakajima W, Ishida A, Ohmura A, Miura S, Takada G (2005). Calpain inhibitor MDL 28170 protects hypoxic-ischemic brain injury in neonatal rats by inhibition of both apoptosis and necrosis. Brain Res.

[26] Northington FJ, Graham EM, Martin LJ (2005). Apoptosis in perinatal hypoxic-ischemic brain injury: how important is it and should it be inhibited?. Brain Res Brain Res Rev.

[27] Stellwagen D, Malenka RC (2006). Synaptic scaling mediated by glial TNF-alpha. Nature.

[28] Perea G, Yang A, Boyden ES, Sur M (2014). Optogenetic astrocyte activation modulates response selectivity of visual cortex neurons in vivo. Nat Commun.

[29] Lopez-Hidalgo M, Schummers J (2014). Cortical maps: a role for astrocytes?. Curr Opin Neurobiol.

[30] Giffard RG, Swanson RA (2005). Ischemia-induced programmed cell death in astrocytes. Glia.

[31] Yu AC, Wong HK, Yung HW, Lau LT (2001). Ischemia-induced apoptosis in primary cultures of astrocytes. Glia.

[32] Wang J, Deng X, Zhang F, Chen D, Ding W (2014). ZnO nanoparticle-induced oxidative stress triggers apoptosis by activating JNK signaling pathway in cultured primary astrocytes. Nanoscale Res Lett.

[33] Xie Z, Smith CJ, Van Eldik LJ (2004). Activated glia induce neuron death via MAP kinase signaling pathways involving JNK and p38. Glia.

[34] Takuma K, Baba A, Matsuda T (2004). Astrocyte apoptosis: implications for neuroprotection. Prog Neurobiol.

[35] Desagher S, Martinou JC (2000). Mitochondria as the central control point of apoptosis. Trends Cell Biol.

[36] Wang X (2001). The expanding role of mitochondria in apoptosis. Genes Dev.

[37] Sun XM, MacFarlane M, Zhuang J, Wolf BB, Green DR, Cohen GM (1999). Distinct caspase cascades are initiated in receptor-mediated and chemical-induced apoptosis. J Biol Chem.

[38] Ferri KF, Kroemer G (2001). Organelle-specific initiation of cell death pathways. Nat Cell Biol.

[39] Nakagawa T, Zhu H, Morishima N (2000). Caspase-12 mediates endoplasmic-reticulum-specific apoptosis and cytotoxicity by amyloid-beta. Nature.

[40] Leist M, Nicotera P (1998). Apoptosis, excitotoxicity, and neuropathology. Exp Cell Res.

[41] Iijima T, Mishima T, Akagawa K, Iwao Y (2003). Mitochondrial hyperpolarization after transient oxygen-glucose deprivation and subsequent apoptosis in cultured rat hippocampal neurons. Brain Res.

[42] Ye R, Li N, Han J (2009). Neuroprotective effects of ginsenoside Rd against oxygen-glucose deprivation in cultured hippocampal neurons. Neurosci Res.

[43] Acehan D, Jiang X, Morgan DG, Heuser JE, Wang X, Akey CW (2002). Three-dimensional structure of the apoptosome: implications for assembly, procaspase-9 binding, and activation. Mol Cell.

[44] Ji YB, Qu ZY, Zou X (2011). Juglone-induced apoptosis in human gastric cancer SGC-7901 cells via the mitochondrial pathway. Exp Toxicol Pathol.

[45] Khan M, Ding C, Rasul A (2012). Isoalantolactone induces reactive oxygen species mediated apoptosis in pancreatic carcinoma PANC-1 cells. Int J Biol Sci.

[46] Wong WW, Puthalakath H (2008). Bcl-2 family proteins: the sentinels of the mitochondrial apoptosis pathway. IUBMB Life.

[47] Moldoveanu T, Follis AV, Kriwacki RW, Green DR (2014). Many players in BCL-2 family affairs. Trends Biochem Sci.

[48] Martinou JC, Youle RJ (2011). Mitochondria in apoptosis: Bcl-2 family members and mitochondrial dynamics. Dev Cell.

[49] Shacka JJ, Roth KA (2005). Regulation of neuronal cell death and neurodegeneration by members of the Bcl-2 family: therapeutic implications. Curr Drug Targets CNS Neurol Disord.

[50] Szegezdi E, Fitzgerald U, Samali A (2003). Caspase-12 and ER-stress-mediated apoptosis: the story so far. Ann NY Acad Sci.

[51] Sergeev IN (2005). Calcium signaling in cancer and vitamin D. J Steroid Biochem Mol Biol.

